# Empirics and the Proposed Veterinary Association in Missouri

**Published:** 1884-07

**Authors:** 


### EDITORIAL NOTES.

EMPIRICS AND THE PROPOSED VETERINARY ASSOCIATION IN MISSOURI.—The correspondent who writes from St. Louis, Mo., calls our attention to the action of the *U. S. Veterinary Journal* in initiating a movement to form a State Association for Missouri, and in accepting, in the call for a convention, the signatures of notorious quacks. He very justly refused to associate himself with others than regular graduates of recognized colleges. He shows, besides, that the time has not yet arrived to form associations in the thinly populated States of the West, and that any attempt to do so at present would be playing into the hands of the empirical members of the profession, who would undoubtedly be the controlling power in them.

We heartily endorse the sentiments expressed by our correspondent, and regret that the journal in question should have proved itself so unworthy of its high mission in failing to protect the veterinary profession from all such influences as tend to impede its onward progress. C.

